# A pilot study of reduced dose cyclosporine and corticosteroids to reduce new onset diabetes mellitus and acute rejection in kidney transplant recipients

**DOI:** 10.1186/2047-1440-2-1

**Published:** 2013-01-12

**Authors:** Edward H Cole, GV Ramesh Prasad, Carl J Cardella, Joseph S Kim, Kathryn J Tinckam, Daniel C Cattran, Jeffrey R Schiff, David N Landsberg, Jeffrey S Zaltzman, John S Gill

**Affiliations:** 1 Toronto General Hospital, 190 Elizabeth Street, M5G 2C4, Toronto, ON, Canada; 2 St Michael's Hospital, 61 Queen Street East, M5C 2T2, Toronto, ON, Canada; 3 Toronto General Hospital, 585 University Avenue, NCSB, M5G 2N2, Toronto, ON, Canada; 4 Histocompatibility Laboratory, 67 College Street, M5G 2M1, Toronto, ON, Canada; 5 St Paul’s Hospital, 1081 Burrard Street, V6Z 1Y6, Vancouver, BC, Canada; 6 St Michael's Hospital, 30 Bond Street, M5B 1W8, Toronto, ON, Canada

**Keywords:** New onset diabetes mellitus, Steroid minimization, Cyclosporine minimization

## Abstract

**Background:**

New onset diabetes mellitus (NODM) and acute rejection (AR) are important causes of morbidity and risk factors for allograft failure after kidney transplantation.

**Methods:**

In this multi-center, open label, single-arm pilot study, 49 adult (≥18 years of age), low immunologic risk, non-diabetic recipients of a first deceased or living donor kidney transplant received early steroid reduction to 5 mg/day combined with Thymoglobulin® (Genzyme Transplant, Cambridge, MA, USA) induction, low dose cyclosporine (2-hour post-dose (C2) target of 600 to 800 ng/ml) and mycophenolic acid (MPA) therapy.

**Results:**

Six months after transplantation, two patients (4%) developed NODM and one patient (2%) developed AR. Four patients had impaired fasting glucose tolerance based on 75-g oral glucose tolerance testing (OGTT). There was one patient death. There were no episodes of cytomegalovirus (CMV) infection or BK virus nephritis. In contrast, in a historical cohort of n = 27 patients treated with Thymoglobulin induction, and conventional doses of cyclosporine and corticosteroids, the incidence of NODM and AR was 18% and 15%.

**Conclusions:**

The pilot study results suggest that Thymoglobulin induction combined with early steroid reduction, reduced cyclosporine exposure and MPA, may reduce the incidence of both NODM and AR in low immunological risk patients. A future controlled study enriched for patients at high risk for NODM is under consideration.

**Trial registration:**

ClinicalTrials.gov: http://NCT00706680

## Background

Despite a high level of awareness of steroid-related side effects, the use of steroid elimination protocols remains limited. A recent registry analysis of data accumulated by the Scientific Registry for Transplant Recipients indicates that only 35% of kidney transplant recipients are now discharged home from hospital without corticosteroids [1]. While short-term results of renal transplantation are excellent, a recent evaluation of longer-term outcomes has shown minimal if any improvement after the first year in the current era [2]. Therefore newer strategies to improve long-term outcomes are required.

New onset diabetes mellitus (NODM) and acute rejection (AR) are important causes of morbidity and risk factors for allograft failure by 5 years [3]. Accordingly, strategies to reduce NODM while maintaining low rates of AR would have the potential to improve long-term outcomes. Recent studies have shown that early steroid withdrawal had limited impact on NODM, but was associated with a higher incidence of AR [4,5]. It is known that NODM is associated with age, ethnicity, hepatitis C, obesity, family history of diabetes and steroids as well as calcineurin inhibitors, especially tacrolimus [6]. Accordingly, it is possible that early steroid withdrawal was not as successful in reducing this metabolic complication as hoped, in part, because of relatively high calcineurin inhibitor doses in one study and the use of tacrolimus in the other [7]. Furthermore, there is also evidence that glucose tolerance is not significantly different among patients treated with low dose prednisone (5 mg/day) and those treated with complete steroid withdrawal [8]. Therefore complete withdrawal (with the potential for more AR) might not be necessary. The main barriers to wider acceptance of steroid elimination are a higher incidence of AR, the absence of clinically relevant improvements in metabolic parameters and hypertension, and concerns about long-term safety.

This pilot study was designed to address the first two concerns. The risk of AR was addressed by the use of Thymoglobulin® induction, and was informed by the findings from the study of Woodle *et al.*[5] where the overall higher rate of AR in the patients withdrawn from corticosteroids compared to those who continued prednisone in doses of 5 mg/day was driven by more AR in the first 3 months after transplantation, but a similar incidence of AR thereafter. We hypothesized that the failure of the study of Woodle *et al.*[5] to demonstrate a clinically relevant improvement in metabolic parameters, such as NODM and kidney function with corticosteroid withdrawal, was due to use of tacrolimus rather than cyclosporine microemulsion, and relatively high tacrolimus target levels after transplantation. Accordingly, we chose to use cyclosporine microemulsion with a lower target based on 2-hour post-dose (C2) monitoring.

This pilot, single-arm study was designed to determine the incidence of NODM and AR with the use of Thymoglobulin, rapid reduction to prednisone 5 mg/day, low-dose cyclosporine and mycophenolic acid (MPA) in low risk patients. By comparing these results with those from a historical cohort of patients, the pilot study would provide information necessary to plan a definitive controlled trial.

## Methods

### Prospective pilot study

#### Study design and sample size

This was an open label, multi-center single-arm pilot study performed in three Canadian transplant centers (Toronto General Hospital, Toronto, St Michael’s Hospital, Toronto, and St Paul’s Hospital, Vancouver) between June 2008 and May 2010. A sample size of 50 patients was planned to provide each center with adequate experience using the immunosuppressive regimen and feasibility including available resources to perform rigorous pre- and post-transplant testing for NODM. The study was approved by the Hospital Research Ethics Board in all three participating hospitals.

### Study population

Adult recipients (≥18 years of age) of a first deceased or living donor kidney only transplant were eligible for the study. Only non-diabetic patients were eligible: all patients were required to have a normal 2-hour 75-g oral glucose tolerance test (OGTT) (<7.8 mmol/l) performed within 1 month of the date of transplantation. Patients with an OGTT of 7.8 to 11.0 mmol/l were classified as having impaired glucose tolerance and were included in the study, while patients with an OGTT >11.0 mmol/l were excluded. In addition, patients were excluded if they had pre-transplant Panel Reactive Antibody >20%, or if they received a zero A, B, DR mismatched kidney. Patients were also excluded if they were unable to provide informed consent or were hepatitis C antibody positive.

### Intervention

The immunosuppressive regimen consisted of Thymoglobulin induction (given pre-operatively or in the early post-operative period, at a dose of 1 to 1.5 mg/kg/day, total dose 4.5 to 7 mg/kg), Neoral cyclosporine (Novartis, Basel, Switzerland)with a target C2 of 600 to 800 ng/ml starting immediately after transplantation, and rapid steroid reduction. All patients received methylprednisolone 250 to 500 mg IV within 1 hour of transplantation followed by prednisone orally at 1 mg/kg/day for 2 days, 0.5 mg/kg/day for 2 days, 0.25 mg/kg/day for 2 days and then 5 mg/day. MPA was administered daily as mycophenolate mofetil (MMF; CellCept, Roche, South San Francisco, CA, USA) 2 g/day, or enteric-coated mycophenolate sodium (EC-MPS; myfortic, Novartis) 1440 mg/day with adjustment as necessary for leucopenia or gastrointestinal symptoms. All patients received prophylaxis against cytomegalovirus (CMV) and *Pneumocystis cainii*, according to center practice. AR was treated per center practice with methylprednisolone IV as first line therapy.

### Outcome

All outcomes were assessed at 6 months. The primary outcome was NODM, which was defined using the following sequential procedure: 1) the continuous use of insulin or oral hypoglycemic medication during follow-up, 2) persistent hyperglycemia defined if >90% of all fasting serum glucose values recorded during routine post-transplant laboratory monitoring in the first 6 months after transplantation were ≥7.0 mmol/l. If neither criteria were met, a 75-gOGTT was performed 6 months after transplantation. Patients with a 2-hour level >11.0 mmol/l were considered diabetic, while patients with an OGTT 7.8 to 11.0 mmol/l were considered to have impaired glucose tolerance.

AR was an important secondary outcome and was determined by biopsy based on Banff 97 criteria, or in circumstances where a biopsy could not be readily obtained, by clinical diagnosis. Other secondary outcomes include patient and allograft survival. Safety outcomes included infections (CMV infection or BK virus nephritis). In addition, reactions to Thymoglobulin infusion (for example hypotension) were documented.

### Historical cohort study

The outcomes of NODM and AR were assessed in a historical cohort of n = 27 non-diabetic patients transplanted at the Toronto General Hospital between January 2000 and December 2007. All patients included in the historical cohort were adult (≥18 years of age) recipients of a first deceased or living donor kidney only transplant, who received Thymoglobulin induction followed by maintenance immunosuppression with standard dose cyclosporine (target C2 level of 1300 to 1500 ng/ml between month 0 to 3 post-transplant, followed by 800 to 1000 ng/ml in months 3 to 6 post-transplant), MPA (the equivalent of MMF 2 g/day), and prednisone (1 mg/kg/day for 3 days, 0.5 mg/kg/day for 4 days, 0.25 mg/kg/day to 1 month, 0.2 mg/kg/day to 2 months and then gradual reduction to 5 mg/day). Patients included in the historical control group were included in the electronic database maintained by the Toronto General Hospital program, which includes information on all prescribed medications and laboratory test results performed for clinical indications before and after transplantation. Patients in the historical control group could not be subjected to the same protocol for diagnosis of diabetes as patients enrolled in the prospective pilot study. Patients were excluded from the historical control group if they had diabetes recorded as a cause of renal failure, were prescribed insulin or oral hypoglycemic medications prior to transplantation, or if they had >1 fasting serum glucose level greater than 7.0 mmol/l within 6 months prior to the date of transplantation. NODM was determined 6 months after transplantation in the historical cohort by insulin or hypoglycemic medication use, or if ≥90% of all fasting glucoses tests were ≥7.0 mmol/l. In addition, patients in the historical cohort were classified as having impaired glucose tolerance if ≥90% of fasting plasma glucose tests were 5.6 to 6.9 mmol/l.

### Analytical methods

Continuous variables were described using the mean and standard deviation (SD), while categorical variables were described using proportions. Outcomes at 6 months after transplantation, including NODM, AR, patient deaths, death-censored transplant failures (including return to dialysis or preemptive repeat transplantation), were described using frequencies.

## Results and discussion

### Prospective pilot study results

Characteristics of the patients enrolled in the prospective pilot study are shown in Table 1. Among the 49 patients enrolled in the pilot study, 96% received living donor allografts, 62% were male, 69% were Caucasian, and there were no African American patients. Prior to transplantation, 38 of 49 patients (78%) were euglycemic by OGTT and 11 patients (22%) had impaired glucose tolerance. Thirty percent had BMI >30 and 82% of patients were unsensitized whereas 18% had a panel reactive antibody (PRA) of 1 to 20%. No patient had a donor-specific antibody at the time of transplantation.

**Table 1:** Characteristics of patients enrolled in prospective pilot study

**Age (mean ±SD)**	**44 ±14 years**
Male	62%
**Race/ethnicity:**	
Caucasian	69%
Indian subcontinent	11%
Other	20%
Missing	0%
**Cause of ESRD:**	
Glomerular	25%
Polycystic disease	18%
Other known	51%
Unknown	6%
Missing	0%
Family history of diabetes	20%
**BMI:**	
Mean ±SD	28 ±5
≤30	70%
>30	30%
Missing	0%
Preemptive transplantation	27%
Hemodialysis	46%
Peritoneal dialysis	27%
Zero PRA (%)	82%
1 to 20% PRA	18%
>20% PRA	0%
Missing	0%
**HLA mismatch:**	
1 to 2%	51%
3 to 6%	49%
Missing	0%
Number of pre-transplant anti-hypertensive medication:	
0	33%
1	31%
>1	26%
**Total cholesterol:**	
<5.2 mmol/l	78%
≥5.2 mmol/l	22%
Living donor	96%
**Pre-transplant 75-gOGTT:**	
Impaired	22%
Normal	78%

BMI, body mass index; ESRD, end stage renal disease; OGTT, oral glucose tolerance test; PRA, panel reactive antibody; SD, standard deviation.

### Protocol adherence

1 of 49 patients was switched from cyclosporine to tacrolimus 1 day post-transplantation. That patient is included in this intent to treat 6-month analysis.

### Primary outcome

#### NODM

One patient died shortly after transplantation and excluded from these analyses. Two of forty-eight patients (4%) developed NODM. Both patients had impaired glucose tolerance prior to transplantation based on 2-hour 75-g OGTT. Four patients (8%) had impaired OGTT at 6 months after transplantation, including two patients (50%) who had impaired glucose tolerance pre-transplant. Forty-two patients (88%) were free of NODM and had normal 2-hour 75-g OGTTs 6 months after transplantation, including seven patients who had impaired glucose tolerance prior to transplantation.

We examined the incidence of NODM in patents at higher risk based on demographic characteristics. Among the n= 11 patients >60 years of age included in the study, 2/11 developed NODM, two had impaired glucose tolerance after the 75-g OGTT and seven had normal glucose levels after the OGTT. In comparison, among the 37 patients ≤60 years of age, none developed NODM, two patients had impaired glucose tolerance after the 75-g OGTT and 35 had normal glucose levels after OGTT. Among the 15 patients with pre-transplant BMI >30, two developed NODM, one patient had impaired glucose tolerance after 75-g OGTT and 12 had normal glucose levels after OGTT. In comparison, among the 33 patients with BMI ≤30, 30 did not develop NODM and three had impaired glucose tolerance. Looking at a combination of age and BMI (Table 2), both patients who developed NODM were >60 years of age and had a BMI >30. Of the four patients with impaired glucose tolerance, two patients were >60 years of age and had BMI ≤30, while two patients were ≤60 years of age, with one having a BMI >30 and one having a BMI ≥30.

**Table 2:** Diabetes status 6 months post-transplant

	**Diabetes**	**Impaired by OGTT**	**Euglycemic**
Age ≤60 years and BMI ≤30 (n = 27)	0	1	26
Age ≤60 years and BMI >30 (n = 10)	0	1	9
Age >60 years and BMI ≤30 (n = 6)	0	2	4
Age >60 years and BMI >30 (n = 5)	2	0	3

BMI, body mass index; OGTT, oral glucose tolerance test.

### Secondary outcomes

AR: One of 48 patients (2%) had a clinically diagnosed episode of AR at 16 days post-transplantation. Serum creatinine in this patient was 149 uM at 6 months, compared to 137 uM at the time of discharge post-transplantation. The episode was treated with steroids alone. This patient had a normal glucose level on the 75-g OGTT test performed 6 months after transplantation.

Patient and allograft survival at 6 months: There was one death 13 days post-transplantation due to severe anoxic brain injury following sepsis. The remaining 48 patients were alive with functioning grafts at the end of study. There were no episodes of CMV infection or BK virus nephritis during follow-up and no infusion reactions to Thymoglobulin were documented.

Blood pressure, kidney function and cholesterol: Mean systolic and diastolic blood pressure (SD) was 135(21)/82 (9) mmHg pre-transplant and 128(11)/78 mmHg at 6 months post-transplant. The mean number of blood pressure medications (SD) was 1.35(1.27) pre-transplant and 1.12(0.91) post-transplant.

At 6 months, the mean ±SD of the serum creatinine was 119 ±39 μM with Modification of Diet in Renal Disease (MDRD) estimated GFR (SD) of 62 (10) ml/min/1.73m^2^. Total fasting cholesterol at 6 months was <5.2 mmol/l in 51% and >5.2 mmol/l in 49%, with an LDL of <2.6 mmol/l in 39% and ≥2.6 mmol/l in 61%. Twenty-seven (55%) patients were not taking a lipid-lowering medication, while 14(30%) were taking one medication, and seven(15%) were taking more than one lipid-lowering medication.

### Historical cohort results

There were a total of n = 27 patients included in the historical cohort who received Thymoglobulin induction and maintenance immunosuppression with conventional doses of cyclosporine and prednisone along with MPA.

The characteristics of the historical control group and comparison with the prospective study patients are shown in Table 3. Among historical controls, the incidence of NODM was 5/27 (18%), while 4/27 (15%) had impaired fasting glucose and 18/27 (67%) had normal fasting glucose measurements. The incidence of AR was 4/27(15%) in this group (Table 3).

**Table 3:** Comparison of prospective study patients with historical control group

	**Prospective study patients**	**Historical control group n = 27**	***P *value**
Age (mean ±SD)	44 ±14 years	49 ±13 years	*P*=0.14
Male	62%	85%	*P*=0.03
**Race/ethnicity:**			
Caucasian	69%	41%	*P*=0.21
Indian subcontinent	11%	4%	
Other	20%	22%	
Missing	0%	33%	
**Cause of ESRD:**			
Glomerular	25%	33%	
Polycystic disease	18%	11%	
Other known cause	51%	41%	
Unknown	6%	15%	*P*=0.41
**BMI:**			
Mean ±SD	28 ±5	29 ±6	*P*=0.57
≤30	70%	56%	*P*=0.23
>30	30%	33%	
Missing	0%	11%	
Preemptive transplantation	27%	37%	*P*=0.34
Living donor	96%	22%	*P*<0.0001
**Diabetes at 6 months:**			
Euglycemic	88%	67%	*P*=0.07
Impaired	8%	15%	
NODAT	4%	18%	
AR at 6 months	2%	15%	*P*=0.033

AR, acute rejection; BMI, body mass index; ESRD, end stage renal disease; NODAT, new onset diabetes after transplantation; SD, standard deviation.

## Discussion

The purpose of this prospective pilot study was to determine whether a strategy of early steroid reduction, but not complete withdrawal, in association with low dose cyclosporine, MPA and Thymoglobulin, would maintain low rates of AR while minimizing the development of NODM. In 48 patients followed for 6 months, there was only one episode of AR, two new cases of NODM (both with impaired glucose tolerance pre-transplant) and two new cases of impaired glucose tolerance. These results compare favorably to those in a historical cohort who in particular had a higher incidence of NODM. We believe that this protocol was successful in minimizing both important metabolic and immunologic complications of transplantation over the short-term.

The benefits of early steroid withdrawal are controversial. In 2009, a large retrospective review of US data showed no reduction in 4-year graft and patient survival in patients discharged from hospital on a steroid-free regimen [9]. Although, in fact graft and patient survival were significantly better in steroid-free patients, the authors comment that the difference was slight and cannot be presumed to be related to steroids because of the retrospective registry-based nature of the study. Another similar retrospective review found that steroids are initiated in a significant percentage of patients initially discharged without them and that this group has lower graft survival than those discharged on steroids [10]. A recent study from the Minneapolis group showed reduced NODM (based on treatment alone), avascular necrosis, cataracts and CMV infection compared to a retrospective control group. However, it should be noted that much higher steroid doses were used in that group compared with practice today, that is 0.4 mg/kg at 1 month and down to 0.15 mg/kg/day at 1 year. Therefore, it is by no means clear that early steroid withdrawal would have these benefits versus the much lower steroid doses used today in steroid-containing protocols. Accordingly, the optimal long-term strategy remains unclear.

Other larger, randomized controlled trials have attempted to maintain freedom from both AR and NODM. The multicenter US study [5] was a randomized double-blind controlled trial over 5 years. In that study, tacrolimus was the calcineurin inhibitor used, steroids were withdrawn completely and some patients received Thymoglobulin, whereas others received anti-CD25 antibodies. Both biopsy-proven AR and chronic allograft nephropathy were more common in the steroid withdrawn group. However, there was no overall difference in NODM based on blood glucose testing although fewer subjects in the steroid withdrawal group needed insulin. There were also no major benefits shown in other cardiovascular risk factors. In the Freedom study, there were 3 groups: standard steroids, steroid withdrawal and a group receiving no steroids. Once again steroid withdrawal (and steroid-free) protocols were associated with significantly more AR. Reduced NODM was only seen in the steroid-free group, which also had the highest AR rate. Of note, two earlier studies did not demonstrate a higher AR rate after steroid withdrawal, but in both cases patients in the control group did not receive induction, potentially accounting for a higher incidence of rejection in those patients [11,12].

The most common causes of late graft loss are patient death and combined interstitial fibrosis and tubular atrophy. Cardiovascular disease is the commonest cause of patient death [13]. Several studies have shown that NODM is associated with both patient death and death-censored graft loss [2,14]. Recent work has demonstrated that NODM occurs in up to 20% of patients in the first year post-transplant [15,16]. It has been suggested, based on retrospective data, which likely underestimates NODM incidence, that NODM is associated with a 90% increase in mortality and 60% increase in graft loss within 3 years post-transplant [15]. Accordingly reduction in NODM is an important goal with the potential to significantly improve long-term graft loss.

Previous studies have shown that tacrolimus is associated with a greater risk of NODM than cyclosporine A [14,17], with similar AR rates when cyclosporine is monitored by C2 [16]. Accordingly we chose cyclosporine as the calcineurin inhibitor in this protocol to minimize NODM risk and antibody induction, to reduce AR risk and permit reduced cyclosporine exposure [4,18]. We chose Thymoglobulin induction based on US data showing a trend towards less AR versus CD25 antibody induction in patients in the early steroid withdrawal arm [5].

While steroid dose reduction would be expected to improve glycemic control, a previous study has shown no additional benefit in glucose control of complete steroid withdrawal below 5 mg/day of prednisone [8]. Accordingly, in an effort to keep AR rates low, we chose to reduce steroids rapidly but to maintain prednisone at 5 mg/day. Our very low incidence of NODM seems superior to that seen by Vincenti *et al*. at 1 year [4], using higher cyclosporine targets, of 14.7% in patients on cyclosporine with standard steroids, or 12.2% in those in whom steroids were withdrawn in 1 week. However, that study included many Agfrican American Black patients, known to be at higher risk of NODM.

The issue of the long-term safety of steroid-sparing regimens is historically linked to the Canadian Multicentre Transplant Study Group [19] because of a higher risk of graft loss in those in whom steroids were discontinued. However, that study had significant methodological problems and the immunosuppression protocols were significantly less effective than those in current use. Despite equivalent graft and patient survival at 5 years in the US study, long-term safety concerns were renewed with findings from a secondary analysis involving a sample of patients with available biopsy data that showed a significantly higher rate of chronic allograft nephropathy in the corticosteroid withdrawal (CSWD) group [5] It was never clarified whether this was attributable to patients with AR in the CSWD group. A recent observational study from the University of Minnesota reported excellent 10-year outcomes with rapid steroid discontinuation and should address the concerns regarding the long-term safety of CSWD at least in Caucasian patients [11].

The major strength of our study is exclusion of diabetic patients from the pilot study and rigorous assessment of NODM after transplantation. The major limitation is a lack of prospective control group and relatively few patients at high risk for NODM based on demographic features. Comparisons with the historical control group reinforce our clinical impression that this regimen safely reduces NODM and AR, and we have continued to use this regimen in clinical practice in low immunological risk patients with high risk of NODM, based on historical features, while we plan a definitive study enriched for patients at greater risk for NODM than those included in this pilot study.

## Conclusions

In summary, a protocol consisting of Thymoglobulin, early steroid reduction to 5 mg/dayof prednisone, low dose cyclosporine dosed by C2 levels, and MPA as immunosuppression resulted in a low rate of both AR and NODM. Thus, this protocol is worthy of further evaluation as a means to preventing these important post-transplant complications.

## Abbreviations

AR: Acute rejection; BMI: Body mass index; C2: Cyclosporine 2-hour post-dose; CMV: Cytomegalovirus; CSWD: Corticosteroid withdrawal; EC-MPS: Enteric-coated mycophenolate sodium; ESRD: End stage renal disease; GFR: Glomerular filtration rate; IV: Intravenous; MDRD: Modification of Diet in Renal Disease; MMF: Mycophenolate mofetil; MPA: Mycophenolic acid; NODM: New onset diabetes mellitus; OGTT: Oral glucose tolerance test; PRA: Plasma renin activity; SD: Standard deviation

## Competing interests

This study was supported by a grant from Genzyme Corporation.

## Authors’ contributions

EC conceived of the study, participated in its design, data analysis and performance of research. GVRP contributed to research design and performance and writing of manuscript. CC contributed to research design and performance and reviewed manuscript. JK participated in performance of research and reviewed manuscript. KT participated in performance of research and reviewed manuscript. DC participated in performance of research and reviewed manuscript. JS participated in performance of research and reviewed manuscript. DL participated in performance of research and reviewed manuscript. JZ participated in performance of research and reviewed manuscript. JG contributed to research design, data analysis, performance of research and writing of manuscript. All authors read and approved the final manuscript.
